# Survival and Growth of Yeast without Telomere Capping by Cdc13 in the Absence of Sgs1, Exo1, and Rad9

**DOI:** 10.1371/journal.pgen.1001072

**Published:** 2010-08-19

**Authors:** Hien-Ping Ngo, David Lydall

**Affiliations:** 1 Institute for Cell and Molecular Biosciences, Newcastle University, Newcastle upon Tyne, United Kingdom; 2 Centre for Integrated Systems Biology of Ageing and Nutrition, Newcastle University, Newcastle upon Tyne, United Kingdom; The University of North Carolina at Chapel Hill, United States of America

## Abstract

Maintenance of telomere capping is absolutely essential to the survival of eukaryotic cells. Telomere capping proteins, such as Cdc13 and POT1, are essential for the viability of budding yeast and mammalian cells, respectively. Here we identify, for the first time, three genetic modifications that allow budding yeast cells to survive without telomere capping by Cdc13. We found that simultaneous inactivation of Sgs1, Exo1, and Rad9, three DNA damage response (DDR) proteins, is sufficient to allow cell division in the absence of Cdc13. Quantitative amplification of ssDNA (QAOS) was used to show that the RecQ helicase Sgs1 plays an important role in the resection of uncapped telomeres, especially in the absence of checkpoint protein Rad9. Strikingly, simultaneous deletion of *SGS1* and the nuclease *EXO1*, further reduces resection at uncapped telomeres and together with deletion of *RAD9* permits cell survival without *CDC13*. Pulsed-field gel electrophoresis studies show that *cdc13-1 rad9Δ sgs1Δ exo1Δ* strains can maintain linear chromosomes despite the absence of telomere capping by Cdc13. However, with continued passage, the telomeres of such strains eventually become short and are maintained by recombination-based mechanisms. Remarkably, *cdc13Δ rad9Δ sgs1Δ exo1Δ* strains, lacking any Cdc13 gene product, are viable and can grow indefinitely. Our work has uncovered a critical role for RecQ helicases in limiting the division of cells with uncapped telomeres, and this may provide one explanation for increased tumorigenesis in human diseases associated with mutations of RecQ helicases. Our results reveal the plasticity of the telomere cap and indicate that the essential role of telomere capping is to counteract specific aspects of the DDR.

## Author Summary

The telomeric DNA of most eukaryotes consists of G-rich repetitive DNA with a 3′ single stranded DNA (ssDNA) overhang. In human and budding yeast (*Saccharomyces cerevisiae*) cells, the 3′ ssDNA overhang is bound by essential telomere capping proteins, POT1 and Cdc13 respectively. Maintenance of telomere capping is essential for the survival of cells. The RecQ helicases are a family of highly conserved proteins involved in the maintenance of telomere and genome stability. Loss of function of three RecQ helicases in humans results in cancer predisposition disorders Bloom's syndrome (BS), Werner's syndrome (WS), and Rothmund Thomson syndrome (RTS). Here we found that the RecQ helicase in budding yeast, Sgs1, plays a critical role in the resection of uncapped telomeres. Strikingly, simultaneous inactivation of Sgs1, the exonuclease Exo1, and checkpoint protein Rad9 allows budding yeast cells to divide in the absence of Cdc13, indicating that the essential role of the telomere cap is to counteract specific components of DNA damage response pathways. We speculate that, in certain genetic contexts, mammalian RecQ helicase also inhibit growth of cells with telomere capping defects, and a defect in this role could contribute to increased levels of tumorigenesis in BS, WS, and RTS patients.

## Introduction

The ends of linear chromosomes pose two major threats to the proliferative potential and genetic stability of eukaryotic cells: inappropriate activation of the DNA damage response (DDR) and progressive shortening of the chromosome ends due to the end replication problem. The telomere, a specialised structure consisting of G-rich repetitive DNA and associated protein complexes at the end of linear chromosomes helps to overcome both of these problems by recruiting telomere capping proteins and telomerase to telomeres [1]. In metazoan organisms, maintenance of telomere integrity is critical for protecting against the processes of cancer and ageing [2], [3].

The mechanisms of chromosome end protection by telomere capping proteins are conserved in eukaryotes. The telomeric DNA of most eukaryotes consists of G-rich repetitive DNA with a 3′ single stranded DNA (ssDNA) overhang. In mammalian cells, telomeric DNA is bound by the shelterin complex [4], [5]. Two proteins of shelterin, TRF1 and TRF2, bind to the double stranded telomeric repeat and recruit TIN2, Rap1, TPP1 and POT1 to the telomere and help to cap the chromosome end [4], [5]. In addition to the shelterin complex, another conserved telomere capping complex, the CST complex, which consists of CTC1, STN1 and TEN1, has been recently described in mammal and plant cells [6]–[8]. In *Saccharomyces cerevisiae*, telomeric ssDNA is capped by the essential Cdc13-Stn1-Ten1 complex, analogous to the CST complex in other cell types [7], [9].

ssDNA binding proteins like POT1 and Cdc13 bind to the telomeric 3′ ssDNA overhang and play multiple roles to protect and maintain the chromosome end [4], [5]. Deletion of *POT1* or *CDC13* results in lethality in both mammalian and yeast cells [10]–[12]. Conditional inactivation of POT1 in mammalian cells leads to telomeric ssDNA generation, ATR-dependent checkpoint activation, deregulation of telomerase, telomere recombination and telomere fusion [4]. Similarly, acute inactivation of Cdc13 by the temperature sensitive *cdc13-1* allele in budding yeast induces telomeric ssDNA generation, recombination and Mec1 (ATR orthologue) dependent cell cycle arrest [10], [13].

The RecQ helicases are a family of highly conserved proteins involved in the maintenance of genome stability and at telomeres [14]. There are five RecQ helicases in humans. Loss of function of three of these results in cancer predisposition disorders Bloom's syndrome (BS, defective in BLM), Werner's syndrome (WS, defective in WRN) and Rothmund Thomson syndrome (RTS, defective in RECQ4) [14]. WS and RTS are also characterised by various features of premature ageing. There is only one RecQ helicase in *Saccharomyces cerevisiae* - *SGS1*. RecQ helicases are 3′-5′ DNA helicases that unwind a variety of DNA replication and recombination structures. It is believed that in the absence of RecQ helicases, genomic rearrangements that arise due to a failure to unwind pathological DNA structures formed at stalled replication forks are a major cause for genetic instability and increases in tumourigenesis associated with these syndromes [14]. The RecQ helicases also play roles in maintaining telomeres and cells derived from WS patients show premature entry into telomere dysfunction-induced senescence, which has been proposed to be a cause for the premature ageing phenotype of WS [15]. Similarly, in yeast, deletion of *SGS1* induces rapid senescence in yeast cell lacking telomerase and Sgs1 is required for the generation of certain type of recombination-dependent ALT (Alternative Lengthening of Telomere) like survivors [16]–[18]. Interestingly, the RecQ helicase in fission yeast, Rqh1 acts on dysfunctional telomeres to promote telomere breakage and entanglement [19].

Several recent studies have identified important roles for the RecQ helicase family in resection of DNA double strand breaks (DSBs) to generate ssDNA, and in generating 3′ overhangs at shortened telomeres [20]–[24]. These resection activities were partially redundant with the 5′ to 3′ exonuclease, Exo1. Since *cdc13-1* induced telomere uncapping leads to telomere resection controlled by Exo1 and other, as yet unidentified, nuclease activities [25], we speculated that Sgs1 might contribute to resection and response to telomere uncapping in *cdc13-1* mutants.

Here we examined the role of Sgs1 in responding to telomere capping defects in *cdc13-1* mutants. We show that although Sgs1 contributes to the good growth of *cdc13-1* mutants, it strongly inhibits the growth of *cdc13-1 exo1Δ rad9Δ* mutants. Our experiments reveal important and yet complex functions of Sgs1 in regulating the growth of budding yeast cells with telomere capping defects. Our results have implications for the role of mammalian RecQ helicases at dysfunctional telomeres.

## Results

### Sgs1, Exo1, and Rad9 affect cell proliferation of *cdc13-1* strains

**Figure 1:**
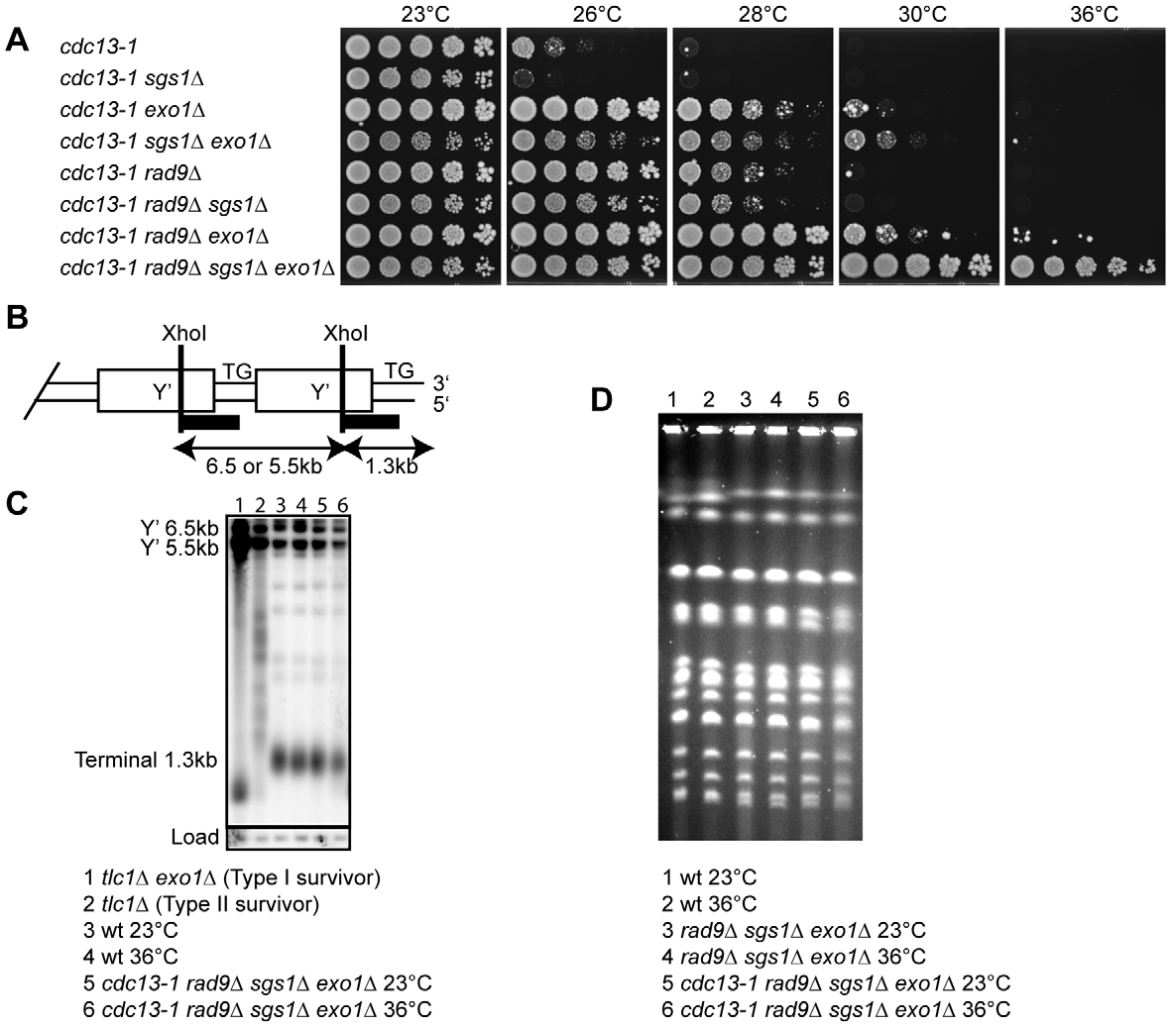
Sgs1, Exo1, and Rad9 affect cell proliferation of *cdc13-1* strains. (A) Serial dilutions of yeast strains with the indicated genotypes and growing at 23°C were spotted onto YPD agar plates and incubated at the indicated temperatures for four days before being photographed. (B) Schematic representation of the end of a Y' repeat-containing yeast chromosome with position of XhoI sites and Southern blot hybridisation probe (black bar) indicated. (C) DNA was purified from the indicated strains following incubation in liquid culture overnight at 23°C or 36°C. The DNA was cut with XhoI and hybridised with a Y'+TG probe as described previously [31]. The membrane was stripped and reprobed with a *CDC15* probe as described previously [31]. (D) Yeast strains with the indicated genotypes were grown in liquid cultures at 23°C or 36°C overnight before subjected to pulsed-field gel electrophoresis as described previously [42].

To begin to test the effect of Sgs1 on the response to telomere uncapping, we first deleted *SGS1* in a *cdc13-1* background. Cdc13-1 becomes increasingly non-functional at temperatures above 23°C, which leads to telomere uncapping, checkpoint activation and temperature sensitive growth [10]. We found that in marked contrast to deletion of *EXO1*, deletion of *SGS1* made *cdc13-1* cells more temperature sensitive at 26°C (Figure 1A). This suggests that Sgs1 contributes to the stability of uncapped telomeres in *cdc13-1* mutants, and that the effect of Sgs1 differs from the effect of Exo1 which attacks uncapped telomeres, generates ssDNA and inhibits growth (Figure 1A) [25]. The role of Sgs1 in stabilising telomeres is consistent with other experiments in budding yeast and mammalian cells [16], [26].

Since Sgs1 functions redundantly with Exo1 to generate ssDNA at DSBs and shortened telomeres [20]–[22], [24], we examined the effect of deleting both Sgs1 and Exo1 activities on growth of *cdc13-1* mutants. We found that *sgs1Δ exo1Δ* mutants grew slightly poorer at all temperatures in both *CDC13* and *cdc13-1* backgrounds (Figure 1A, Figure S1). The poor growth of *CDC13 sgs1Δ exo1Δ* mutants has been reported previously [20], [21]. Despite the poor growth of *sgs1Δ exo1Δ* mutants, deletion of *SGS1* allowed *cdc13-1 exo1Δ* strains to grow slightly better at high temperature, since more cells appeared able to divide and grow at 30°C (Figure 1A). This suggests that in the absence of Exo1, Sgs1 may play a role inhibiting cell division when telomeres uncap.

Checkpoint pathways, as well as nucleases, inhibit the growth of *cdc13-1* cells at high temperature [25], [27]. We have previously shown that simultaneous deletion of *EXO1* and the checkpoint gene *RAD9* allow better suppression of *cdc13-1* temperature sensitivity than either single deletion [25]. Strikingly, deletion of *SGS1* fully suppressed the temperature sensitivity of *cdc13-1 rad9Δ exo1Δ* strains and allowed *cdc13-1 rad9Δ sgs1Δ exo1Δ* strains to form colonies at 36°C, a temperature at which Cdc13 is expected to be completely non-functional (Figure 1A, Figure S2) [10]. This suggests that deletion of *SGS1*, *EXO1* and *RAD9* allows budding yeast cells to grow in the absence of any Cdc13 activity.

It was possible that growth of *cdc13-1 rad9Δ sgs1Δ exo1Δ* strains at 36°C was due to second site mutations/changes since we previously showed that at low rates (5×10^−5^) *cdc13-1 exo1Δ rad9Δ* strains could generate clones that can grow at 36°C [28]. Therefore to test the possibility that a second site mutation was responsible for the growth of *cdc13-1 rad9Δ sgs1Δ exo1Δ* strains at 36°C, we performed a backcross with an independent *cdc13-1* strain. We found that all *cdc13-1 rad9Δ sgs1Δ exo1Δ* segregants derived from the cross were able to grow at 36°C (Figure S3). This demonstrated that no independently segregating mutation contributed to growth of *cdc13-1 rad9Δ sgs1Δ exo1Δ* strains at high temperature, and suggests that Sgs1, Exo1 and Rad9-dependent activities combine to inhibit the growth of telomere capping defective *cdc13-1* mutants at 36°C.

At low rates a small fraction of telomerase-null ALT survivors that maintain telomeres by homologous recombination have also been shown to be able to survive without Cdc13 [29]. Therefore to test whether alterations in telomere structure were responsible for the good growth of *cdc13-1 rad9Δ sgs1Δ exo1Δ* strains at 36°C, we examined telomere structure by Southern blot. As positive controls, we examined both Type I and Type II telomerase-null ALT survivors which have amplified the Y' region and the TG repeats respectively. We found that *cdc13-1 rad9Δ sgs1Δ exo1Δ* strains had telomere structures that looked similar to wild-type strains at both 23°C and 36°C (Figure 1B, Figure 1C and data not shown). Furthermore, we found that *cdc13-1 rad9Δ sgs1Δ exo1Δ* strains can grow and maintain normal telomere structure at 36°C without Rad52, suggesting that homologous recombination is not required for the survival of these strains following telomere uncapping (Figure S4). Therefore we conclude that *cdc13-1 rad9Δ sgs1Δ exo1Δ* strains are not ALT survivors and the growth at 36°C is independent of major alterations in telomeric DNA structure.

In *Schizosaccharomyces pombe*, inactivation of the telomere capping protein Pot1 results in growth crisis and generation of survivors with circular chromosomes [30]. To test whether *cdc13-1 rad9Δ sgs1Δ exo1Δ* strains survive growth at 36°C by circularising their chromosomes, we used pulsed-field gel electrophoresis to examine chromosome structure. Circular chromosomes do not enter pulsed field gels. We found that at 23°C, *cdc13-1 rad9Δ sgs1Δ exo1Δ* strains contain linear chromosomes that were indistinguishable from wild-type or *rad9Δ sgs1Δ exo1Δ* strains (Figure 1D). Interestingly, the chromosomes of *cdc13-1 rad9Δ sgs1Δ exo1Δ* strain remain linear at 36°C (Figure 1D). We conclude that the ability of *cdc13-1 rad9Δ sgs1Δ exo1Δ* strains to grow without telomere capping by Cdc13 is not due to chromosome circularisation.

The Mre11-Rad50-Xrs2 (MRX) complex and the nuclease Sae2 can resect DSBs in an Sgs1 and Exo1-independent pathway [20], [22]. To test whether inhibition of this pathway also has an effect on response to telomere uncapping and ability of *cdc13-1* cells to grow at 36°C, we examined the effect of deleting *SAE2* on growth of *cdc13-1* mutants. We found that like the MRX complex [31], Sae2 has a role in the protection of uncapped telomeres as deletion of *SAE2* renders *cdc13-1* cells slightly more temperature sensitive at 26°C (Figure S5). Furthermore, deletion of *SAE2*, in combination with deletion of *SGS1 or EXO1* in the presence or absence of *RAD9* does not allow *cdc13-1* cells to grow at 36°C (Figure S5). We were unable to analyse *sgs1Δ sae2Δ exo1Δ* triple mutant cells as simultaneous deletion of these genes is lethal, as reported previously [22], [24] (Figure S6). We conclude that Sae2 has a protective role at uncapped telomeres and that unlike Sgs1, deletion of *SAE2* does not allow *cdc13-1 rad9Δ exo1Δ* cells to grow at 36°C.

### Checkpoint response to telomere uncapping in the absence of Sgs1, Exo1, and Rad9

**Figure 2:**
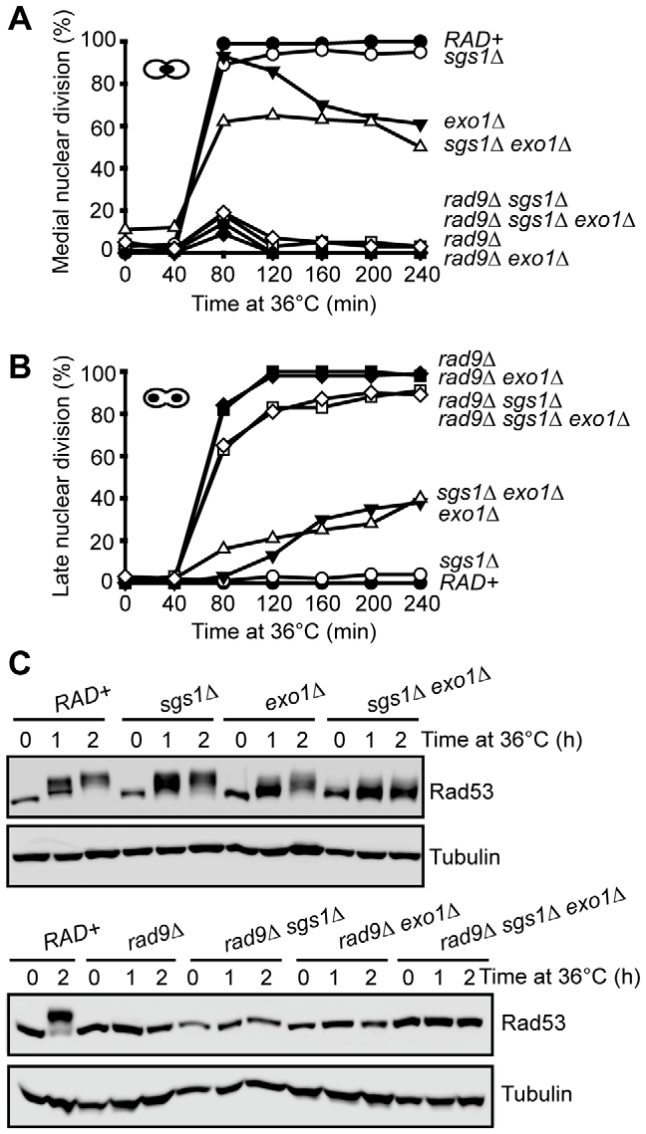
Checkpoint response to telomere uncapping in the absence of Sgs1, Exo1, and Rad9. (A) Yeast strains of the indicated genotypes (all with *cdc13-1 cdc15-2 bar1* mutations) were arrested in G1 at 23°C with α factor and released into 36°C, cells were collected at the indicated time points, and scored for the percentage of cells arrested in medial nuclear division using DAPI staining. (B) As in (A) but the percentage of cells arrested in late nuclear division were quantified. (C) Yeast strains of the indicated genotypes (all with *cdc13-1 cdc15-2 bar1* mutations) were arrested in G1 at 23°C and released into 36°C for up to two hours. Western blot were first probed with anti-Rad53 antibody, membranes were stripped and reprobed with anti-tubulin antibody.

To further address the surprising ability of *cdc13-1 rad9Δ sgs1Δ exo1Δ* strains to grow at 36°C, we used synchronous cultures to examine two cellular responses to telomere uncapping: checkpoint activation and telomeric ssDNA generation. To examine checkpoint activation, we first quantified the fraction of cells arrested at medial nuclear division induced by *cdc13-1* mutation at 36°C. A *cdc15-2* mutation, which inhibits mitotic exit, was used to inhibit further cell cycle progression of any *cdc13-1* cells that failed to arrest at medial nuclear division or escaped arrest and therefore progressed to reach the late nuclear division stage [27]. As expected, control *cdc13-1* cells were largely arrested at medial nuclear division 80 minutes after release from G1 arrest to 36°C and the cells remained arrested for up to 4 hours (Figure 2A and 2B) [25]. A *cdc13-1 sgs1Δ* strain showed a similar cell cycle arrest to the *cdc13-1* strain (Figure 2A and 2B). *cdc13-1 exo1Δ* strains showed no defect in the activation of checkpoint as most *cdc13-1 exo1Δ* cells were arrested at medial nuclear division after 80 minutes, but some cells escaped from the arrest at later time points, as previously reported (Figure 2A and 2B) [25]. We found that deletion of both *SGS1* and *EXO1* slightly reduced the number of cells arrested at medial nuclear division (to approximately 60%) after 80 minutes (Figure 2A). This is partly due to a slower cell cycle progression and also due to a mild defect in checkpoint activation as approximately 15% of *cdc13-1 sgs1Δ exo1Δ* cells had entered late nuclear division by 80 minutes (Figure 2B, Figure S7). At later time points, increasing fractions of *cdc13-1 sgs1Δ exo1Δ* cells passed through medial nuclear division and accumulated at the late nuclear division stage, presumably due to a defect in either activation or maintenance of checkpoint (Figure 2B). As expected, deletion of *RAD9* completely eliminated checkpoint arrest in all these backgrounds (Figure 2A and 2B). We conclude that in the absence of both Sgs1 and Exo1, checkpoint activation/maintenance following telomere uncapping in *cdc13-1* mutants is partially defective.

We next examined Rad53 mobility shifts, caused by checkpoint-kinase-dependent phosphorylation, as a biochemical marker for checkpoint activation in *cdc13-1* strains [32]. It has been shown that Rad53 is strongly phosphorylated two hours following telomere uncapping in *cdc13-1* mutants [32]. Consistent with this finding, we observed a strong Rad53 phosphorylation shift two hours after *cdc13-1* cells were shifted to 36°C (Figure 2C). Deletion of *SGS1* in *cdc13-1* strains had little effect on this Rad53 phosphorylation but deletion of *EXO1* mildly reduced Rad53 phosphorylation (Figure 2C). Interestingly, we found that deletion of both *SGS1* and *EXO1* caused a more severe defect on Rad53 phosphorylation, although some residual Rad53 phosphorylation could be detected (Figure 2C). As expected, Rad53 phosphorylation was abolished when *RAD9* was deleted in all strains (Figure 2C). Taken together, the cell cycle arrest and Rad53 phosphorylation experiments suggest that Sgs1 and Exo1 control parallel pathways to activate the checkpoint induced by uncapped telomeres. However, the presence of residual Rad53 phosphorylation and checkpoint arrest in *cdc13-1 sgs1Δ exo1Δ* strains indicates that there is at least one additional pathway to activate the checkpoint cascade after telomere uncapping in the absence of Sgs1 and Exo1.

### Resection of uncapped telomeres in the absence of Sgs1, Exo1, and Rad9

**Figure 3:**
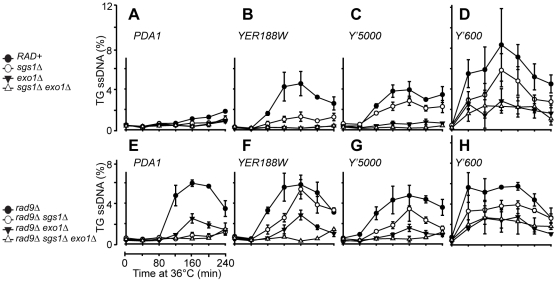
Resection of uncapped telomeres in the absence of Sgs1, Exo1, and Rad9. (A–H) A series of yeast strains with the indicated genotypes (all with *cdc13-1 cdc15-2 bar1* mutations) were arrested in G1 at 23°C and released into 36°C to induce telomere uncapping, the amount of ssDNA at the TG strands at two repetitive telomeric locus, Y'600 and Y'5000 and two single copy loci, *YER188W* and *PDA1* were measured by QAOS as described previously [41]. The values plotted are the mean value ± SD.

The major stimulus for checkpoint cascades after telomere uncapping is ssDNA accumulation caused by 5′ to 3′ resection, generating ssDNA specifically on the TG rich, 3′ strand at telomeres [25], [33]. To study the role of Sgs1 in resection, we used Quantitative amplification of ssDNA (QAOS) [25], [33] to examine ssDNA production at two repetitive loci in subtelomeric regions and three single copy loci near the right telomere of chromosome V. We first examined the Y' repeats which are found in two thirds of yeast telomeres. We measured ssDNA accumulation at two different Y' loci, *Y'600* and *Y'5000*, located approximately 600 bp and 5000 bp from many chromosome ends. As previously reported, we detected accumulation of ssDNA specifically on the TG strands and this ss DNA was partly dependent on Exo1 (Figure 3C and 3D, Figure S8) [25]. This ssDNA was also partly dependent on Sgs1, this was particularly clear at the *Y'5000* locus (Figure 3C). The difference between ssDNA accumulation in *cdc13-1* and *cdc13-1 sgs1Δ* cells was perhaps not as significant at *Y'600* as some standard deviations overlapped (Figure 3D). Interestingly, in the absence of both Sgs1 and Exo1, telomere resection was not detectable at *Y'5000*, but was still detectable at *Y'600* (Figure 3C and 3D, Figure S9). This suggests that Sgs1 and Exo1 independently regulate 5′-3′ resection at *cdc13-1*-induced uncapped telomeres, especially further away from the telomere ends at *Y'5000* loci. This resection defect likely provides an explanation to the weak checkpoint activation observed in *cdc13-1 sgs1Δ exo1Δ* strains (Figure 2, Figure S7). At *Y'600*, the ssDNA generated in *cdc13-1 sgs1Δ exo1Δ* cells was not significantly different from that seen in *cdc13-1 exo1Δ* cells (Figure 3D). The presence of ssDNA at *Y'600* loci in *cdc13-1 sgs1Δ exo1Δ* strains suggests that there is a third pathway that can resect uncapped telomeres near the telomere ends (Figure 3D). The small residual amounts of telomeric ssDNA likely provide the stimulus for checkpoint activation in *cdc13-1 sgs1Δ exo1Δ* strains.

It has been shown that following telomere uncapping in *cdc13-1* mutants, the resection of uncapped telomeres extends into the single copy regions of chromosomes [10]. To test whether Sgs1 affects accumulation of ssDNA in single copy regions, we examined ssDNA accumulation further away from the telomeres at three single copy loci, *YER188W*, *YER186C* and *PDA1* located at 8.5 kb, 14.5 kb and 29.7 kb from the right end of Chromosome V (Figure 3A and 3B, Figure S8). As previously observed, we detected ssDNA in *cdc13-1* cells at both the *YER188W*, *YER186C* loci, but not at the *PDA1* locus which is located further away from the telomeres (Figure 3A and 3B, Figure S8). We found that the resection at these loci were highly dependent on *EXO1* as previously reported [25], but considerably less dependent on Sgs1 (Figure 3A and 3B, Figure S8). Thus our data suggests that Sgs1 and Exo1 regulate different types of nuclease activities in *cdc13-1* mutants.

We next examined resection in the absence of *RAD9* because Rad9 plays an important role in the inhibition of nuclease activities at uncapped telomeres [25], [27], [34]. Specifically, deletion of *RAD9* allows nuclease(s) activities to generate ssDNA further away from the telomere ends, even in the absence of Exo1, which results in the resection of DNA up to 30 kb away from the telomeres [25]. We first examined ssDNA production at the Y' repeats and found that as in *RAD9+* background, DNA resection of uncapped telomeres at the *Y'5000* loci was totally dependent on the combined activities of Sgs1 and Exo1 as little ssDNA was detected at these loci following telomere uncapping (Figure 3G, Figure S8). This observation again supports the idea that Sgs1 and Exo1 act in two alternative pathways to resect the DNA at these loci. Consistent with what was observed in *RAD9+* background, there appears to be another nuclease that can resect the uncapped telomeres near the telomere end at Y'600 in the absence of both Sgs1 and Exo1 (Figure 3H). As previously reported, we found that deletion of *RAD9* allows ssDNA generation at single copy loci up to 30 kb away from the telomere ends even in the absence of Exo1 (compare *cdc13-1 exo1Δ* in Figure 3A and 3B and *cdc13-1 rad9Δ exo1Δ* in Figure 3E and 3F). Interestingly, deleting *SGS1* abolished this Exo1-independent ssDNA generation (compare *cdc13-1 rad9Δ exo1Δ* and *cdc13-1 rad9Δ sgs1Δ exo1Δ*) (Figure 3E and 3F). This suggests that Rad9 inhibits an Sgs1-dependent, but Exo1-independent pathway of generating ssDNA further away from the uncapped telomere ends.

The results described above suggest that Sgs1 and Exo1 contribute to two distinct pathways of resection near the telomeres of *cdc13-1* strains. Our data suggests that attenuation of telomeric DNA resection pathways by deletion of both *SGS1* and *EXO1*, coupled with inactivation of checkpoint machinery by deletion of *RAD9*, is sufficient to allow cells to grow in the absence of telomere capping by Cdc13.

### Cellular response to long term absence of Cdc13

**Figure 4:**
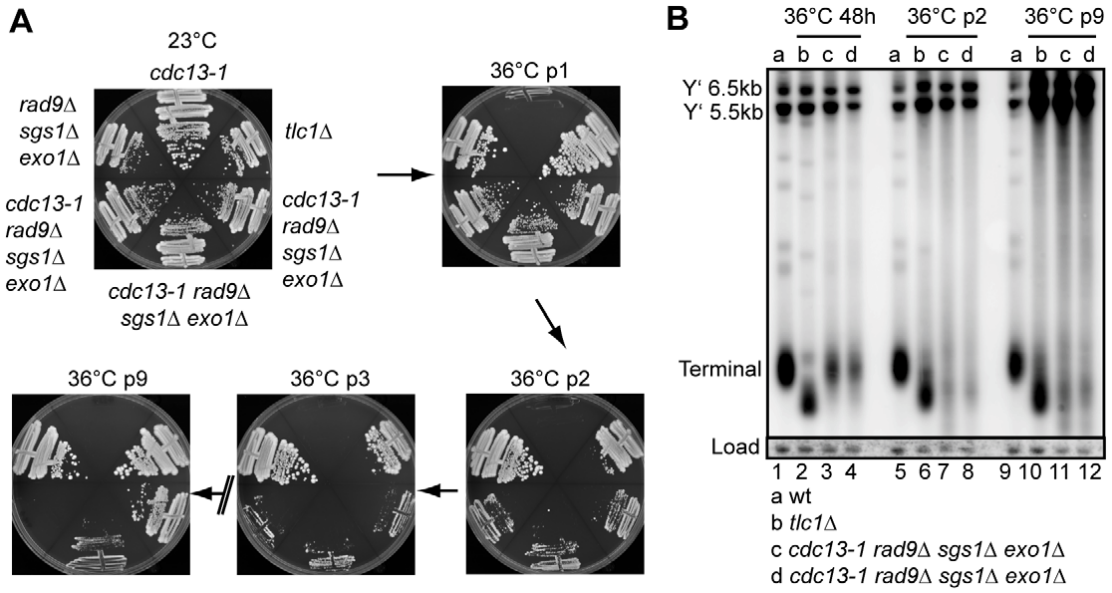
Cellular response to long term absence of Cdc13. (A) Yeast strains of the indicated genotype were streaked onto YPD agar plates and the plates were incubated at 23°C or 36°C for three days. The strains were then photographed and restreaked onto another YPD plate. This cycle is repeated for nine times (p = passage). (B) DNA was purified from the strains in (A) or a wild-type strain following further incubation in liquid culture for 48 hours at 36°C. Southern blots were performed as in Figure 1C.

Cdc13 plays at least two important roles at telomeres, one capping chromosome ends, the other recruiting telomerase [35]. *cdc13-1* strains grown at 36°C would be expected to be defective in both activities. Therefore to test whether *cdc13-1 rad9Δ sgs1Δ exo1Δ* strains can maintain telomeres and/or tolerate long term absence of Cdc13, we grew cells for many passages at 36°C by repeatedly restreaking them onto plates which were incubated at 36°C (Figure 4A). We found that *cdc13-1 rad9Δ sgs1Δ exo1Δ* strains showed progressively slower growth with repeated passage at 36°C, but that some strains eventually accumulated faster growing colonies (Figure 4A). This pattern of growth is similar to telomerase deficient *tlc1Δ* cells which enter growth senescence due to shortened telomeres, and eventually accumulate post senescent survivors which survive by utilising recombination-dependent mechanisms to maintain their telomeres (Figure 4A) [36], [37].

To test whether *cdc13-1 rad9Δ sgs1Δ exo1Δ* strains growing at 36°C were behaving like telomerase deficient strains, we examined the telomere structure by Southern blot. At early passage, *cdc13-1 rad9Δ sgs1Δ exo1Δ* strains grown at 36°C had a comparatively normal telomeric DNA structure, but the terminal telomere fragments were weak (Figure 4B, Figure 1C). This confirms that growth of *cdc13-1 rad9Δ sgs1Δ exo1Δ* strains at 36°C does not depend on gross changes in telomere structure. By passage 2, the terminal telomere fragments of these strains had become shorter and weaker, similar to but distinct from the telomeres in a *tlc1Δ* strain, possibly explaining the poor growth of these strains at this passage at 36°C (Figure 4A and 4B). At passage 9, when some of the *cdc13-1 rad9Δ sgs1Δ exo1Δ* strains grew well they showed alternative telomere structures similar to that in a Type I *tlc1Δ* survivor and had amplified the 5.5 and 6.5 kb Y' repeats (Lanes 10-12, Figure 4B) [36]. Unlike the Type I *tlc1Δ* survivor shown, the terminal fragments in *cdc13-1 rad9Δ sgs1Δ exo1Δ* survivors remained very weak. We conclude that initial growth of *cdc13-1 rad9Δ sgs1Δ exo1Δ* strains at 36°C does not depend on gross alteration in telomere structure. However, continued growth without functional Cdc13 results in telomere shortening, cellular senescence and generation of post-senescence survivors with altered telomere structures.

### Cdc13 is dispensable for cell viability in the absence of Sgs1, Exo1, and Rad9

**Figure 5:**
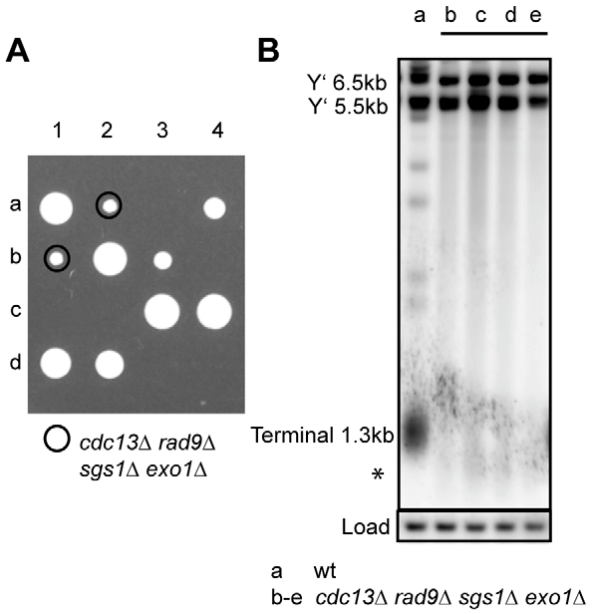
Cdc13 is dispensable for cell viability in the absence of Sgs1, Exo1, and Rad9. (A) Spores from a yeast strain heterozygous for *cdc13Δ*, *sgs1Δ*, *exo1Δ* and *rad9Δ* were dissected onto YPD agar plate and the plates were incubated at 23°C for five days before being photographed. Four tetrads (labelled 1–4) are shown (see Figure S10 for full genotype). (B) DNA was purified from fresh *cdc13Δ sgs1Δ exo1Δ rad9Δ* strains or control strains following further incubation in liquid culture for 48 hours at 23°C. Southern blots were performed as in Figure 1C.

The growth of *cdc13-1 rad9Δ sgs1Δ exo1Δ* strains at 36°C suggested that *CDC13* might not be essential in cells lacking *SGS1, EXO1* and *RAD9*. To directly test this hypothesis, we created diploid strains heterozygous for *sgs1Δ*, *exo1Δ*, *rad9Δ* and *cdc13Δ* and dissected tetrads following sporulation (Figure 5A, Figure S10). We found that *cdc13Δ rad9Δ sgs1Δ exo1Δ* spores were viable and formed colonies at a comparable frequency (average 89%) to *CDC13 rad9Δ sgs1Δ exo1Δ* spores, whereas other *cdc13Δ* genotypes were unviable (Figure 5A, Figure S10, Table S1). This result shows that deleting *SGS1, EXO1* and *RAD9* genes from yeast is sufficient to allow yeast cells to grow and divide in the absence of the usually essential telomere capping protein Cdc13. We found that the telomeres of *cdc13Δ rad9Δ sgs1Δ exo1Δ* strains shortened very quickly and the terminal fragments of fresh *cdc13Δ rad9Δ sgs1Δ exo1Δ* strains (marked with asterisk) were already shortened when first examined (Figure 5B). Similar to *cdc13-1 rad9Δ sgs1Δ exo1Δ* strains at 36°C, these terminal fragments of *cdc13Δ rad9Δ sgs1Δ exo1Δ* strains were very weak (Figure 5B). We found that these *cdc13Δ rad9Δ sgs1Δ exo1Δ* cells show variable rates of senescence, and faster growing ALT survivors that can grow for at least eight passages without Cdc13 eventually emerged (Figure S11, Figure S12). We conclude that Cdc13 is not an essential telomere capping protein in *rad9Δ sgs1Δ exo1Δ* strains, but that such strains eventually maintain telomeres like telomerase deficient cells.

## Discussion

Telomere capping protects the ends of linear chromosomes from the activation of DDR. However, cell division-induced telomere shortening in human somatic cells is believed to lead to progressive loss of telomere capping, and causes chromosome ends to be recognised as DNA breaks, which consequently activate a DDR and cell cycle arrest [4]. This telomere dysfunction-induced cell cycle arrest is thought to contribute to the establishment of replicative cellular senescence and the ageing process, and represent an important barrier to tumour formation [2], [38], [39]. Thus it is important to understand the cellular response to telomere uncapping.

Budding yeast temperature sensitive *cdc13-1* mutants have been an informative model to understand the eukaryotic cell response to telomere uncapping. It was shown previously that at low rates *cdc13-1 rad9Δ exo1Δ* cells (5×10^−5^) or telomerase deficient survivors (8×10^−5^ to 4×10^−2^) were able to grow without telomere capping by Cdc13, but the nature of any other genetic or other change necessary to permit growth was unclear [28], [29]. Here we show that the simultaneous deletion of *SGS1*, *EXO1* and *RAD9* is sufficient to make Cdc13 dispensable for cell proliferation, as most *cdc13Δ rad9Δ sgs1Δ exo1Δ* cells are viable. This indicates that the essential function of telomere capping in yeast is to counteract the proliferative barrier exerted by specific components of the DDR. Our data suggests that attenuation of resection stimulating activities of Sgs1 and Exo1, together with DNA damage checkpoint inactivation is sufficient to allow yeast cells to grow in the absence of telomere capping by Cdc13. This suggests that in the absence of these proteins, either telomere capping is not required or that an alternative capping strategy can function in the absence of Cdc13. Intriguingly, the telomeres in *cdc13-1 rad9Δ sgs1Δ exo1Δ* strains (at 36°C) and *cdc13Δ rad9Δ sgs1Δ exo1Δ* strains look different to those in wild-type strains, as the telomeres have very weak terminal restriction fragments. Even though we showed that *cdc13-1 rad9Δ sgs1Δ exo1Δ* strains do not require Rad52 to survive telomere uncapping at 36°C, we cannot rule out a role for homologous recombination proteins at telomeres in *cdc13Δ rad9Δ sgs1Δ exo1Δ* strains.

Besides capping telomeres, Cdc13 is also required for the recruitment of telomerase [35], and as expected we found that in the absence of Cdc13, telomeres eventually shorten and are then maintained by the alternative lengthening of telomere (ALT) mechanisms, usually seen in telomerase deficient cells. Our work highlights the plasticity of the telomere cap and shows how modulation of the DDR can provide an important mechanism to overcome the proliferative barrier induced by dysfunctional telomeres. As telomere uncapping-induced cellular senescence represents an important proliferative barrier to prevent cancer formation, the mechanisms described here could be relevant to understanding the malignant transformation of human cells [2], [3].

The RecQ helicases, including Sgs1, are a family of highly conserved protein involved in the maintenance of genome stability and suppression of cancer formation in humans [14]. It is believed that in the absence of RecQ helicases, genome rearrangements that arise at stalled replication forks are a major cause for the increased tumorigenesis [14]. RecQ helicases also participate in the maintenance of telomeres, for example, deletion of *SGS1* induces rapid senescence in yeast cells lacking telomerase and cells derived from WS patients show premature entry into telomere dysfunction-induced senescence [15], [16]. Here we report a similar role for Sgs1 in the protection of uncapped telomeres as deleting *SGS1* makes *cdc13-1* cells more temperature sensitive. However, paradoxically, we also show that Sgs1 plays a role in the resection of uncapped telomeres, an activity that is modulated by the checkpoint protein Rad9. This Sgs1-dependent resection activity leads to a critical role of Sgs1 in the inhibition of cell proliferation when telomeres uncap in the absence of Exo1 and Rad9. It is tempting to speculate that in certain genetic contexts RecQ helicase may also inhibit growth of mammalian cells with telomere capping defects, and a defect in this role could contribute to increased levels of tumorigenesis in BS, WS and RTS patients.

Previous studies have suggested that following telomere uncapping in *cdc13-1* mutants, the telomeres were resected by three somewhat redundant nuclease activities, Exo1, ExoX and ExoY [25]. ExoX was defined as a Rad9-inhibited nuclease that was strongly dependent on Rad24 [25]. ExoY was a nuclease that acts on uncapped telomeres in the absence of Exo1 and Rad24 (ExoX). In this study, we have identified Sgs1 as a protein that shares similar properties with ExoX, because it is required for the resection of uncapped telomeres in an Exo1-independent pathway and its activity is inhibited by Rad9. Since the helicase Sgs1 acts with a nuclease Dna2 in the resection of DSBs and at shortened telomeres [20], [24], we speculate that Sgs1 and Dna2 might work together to regulate resection at uncapped telomeres (may contribute to ExoX). We also found that in the absence of Sgs1 and Exo1, ssDNA still accumulated at *cdc13-1* induced uncapped telomeres, consistent with the existence of another nuclease (possibly ExoY). It is interesting to note that the Mre11-Rad50-Xrs2 (MRX) complex and the nuclease Sae2 can resect DSBs in an Sgs1 and Exo1-independent pathway [20], [22]. Thus a candidate for ExoY is MRX/Sae2. However, we have previously found that the MRX complex plays a capping role at telomeres, rather than contributing to resection [31]. Similarly, we found that Sae2 also has a protective role at uncapped telomeres. Therefore we believe that like the MRX complex, Sae2 is unlikely to be ExoY. Thus although uncapped telomeres share significant similarities with DSBs, there are also clearly significant differences. Future studies will be required to resolve the roles of the different types of nucleases and nuclease regulators at uncapped telomeres and DSBs.

## Materials and Methods

### Yeast strains

All experiments were performed using *Saccharomyces cerevisiae* W303 strains as listed in Table S2. Gene disruptions of *SGS1* and *CDC13* were constructed by a one step PCR-mediated method using kanMX and hphMX cassettes respectively [40]. All the yeast strains were generated by standard genetic crosses.

### Spot tests

Single colonies were inoculated into 2 ml YPD and incubated at 23 °C overnight until saturation. Five-fold serial dilution of the cultures were spotted onto agar plates using a 48 or 96-prong replica plating device. Plates were incubated for 2–4 days at different temperatures before being photographed using a SPimager (S&P Robotics).

### Protein extraction and western blot analysis

Protein extracts were prepared by using TCA extraction method, as previously described [32]. Briefly, cells were collected, washed in water, resuspended in 10% TCA and broken using glass beads. Protein suspensions were then resuspended in Laemmli buffer. Samples were boiled for three minutes, centrifuged for 10 minutes and the supernatant used as the protein extract. For Western blotting, proteins were separated on 7.5% SDS-PAGE. Anti-Rad53 and anti-tubulin antibodies were from Dan Durocher, Toronto and Keith Gull, Oxford respectively.

### Analysis of telomere structure

DNA was purified from yeast strains following incubation in liquid culture for 24 or 48 hours. The DNA was cut with XhoI, run on a 0.8% (0.5xTBE) gel at 1V/cm overnight and transferred to a Magna nylon membrane. The membrane was then hybridised with a Y'+TG probe (synthesized using DIG-High Prime Labelling and Detection Kit (Roche)) as described previously [31]. The membrane was stripped and reprobed with a *CDC15* probe as described previously [31].

### Cell cycle analysis and single-stranded DNA measurement

Yeast strains with *cdc13-1 cdc15-2 bar1* mutations were arrested in G1 at 23°C and released into 36°C to induce telomere uncapping, cell cycle position were scored using DAPI staining on a Nikon Eclipse 50i microscope. The amount of ssDNA at the TG and the AC strands at telomeric and single copy loci were measured by QAOS (quantitative amplification of single-stranded DNA) as described previously [41].

### Pulsed-field gel electrophoresis

Pulsed-field gel electrophoresis were carried out as described previously with the following modification: gels were run at 6.0 V/cm with a switch time of 70 seconds for 15 hours and 120 seconds for 11 hours [42].

## Supporting Information

Figure S1 Growth of *CDC13* strains at different temperatures. Serial dilutions of yeast strains with the indicated genotypes and growing at 23°C were spotted onto the same YPD agar plates in Figure 1A and incubated at the indicated temperatures for four days before being photographed.
(1.98 MB PDF)

Figure S2 *cdc13-1 rad9Δ sgs1Δ exo1Δ* strains show little sensitivity to telomere uncapping at 36°C. Serial dilutions of yeast strains with the indicated genotypes and growing at 23°C were spotted onto YPD agar plates and incubated at the indicated temperatures for three days before being photographed.
(3.90 MB PDF)

Figure S3 Growth of *cdc13-1 rad9Δ sgs1Δ exo1Δ* strains at 36°C is not due to another mutation. Serial dilutions of yeast strains with the indicated genotypes and growing at 23°C were spotted onto YPD agar plates and incubated at the indicated temperatures for three days before being photographed.
(5.75 MB PDF)

Figure S4 Growth of *cdc13-1 rad9Δ sgs1Δ exo1Δ* strains at 36°C is not dependant on Rad52. (A) Serial dilutions of yeast strains with the indicated genotypes and growing at 23°C were spotted onto YPD agar plates and incubated at the indicated temperatures for three days before being photographed. (B) DNA was purified from the strains with the indicated genotypes following further incubation in liquid culture overnight at 23°C or 36°C. Southern blots were performed as in Figure 1C.
(5.79 MB PDF)

Figure S5 Genetic interactions of *sae2Δ* with *sgs1Δ*, *exo1Δ*, *rad9Δ* and *cdc13-1*. Serial dilutions of yeast strains with the indicated genotypes and growing at 23°C were spotted onto YPD agar plates and incubated at the indicated temperatures for three days before being photographed. The top panel show genetic interaction of *sae2Δ* with *exo1Δ*, *rad9Δ* and *cdc13-1* whereas the bottom panel show genetic interaction of *sae2Δ* with *sgs1Δ*, *rad9Δ* and *cdc13-1*.
(9.47 MB PDF)

Figure S6 Genotypes of spores from a yeast strain heterozygous for *cdc13-1*, *sgs1Δ*, *exo1Δ, sae2Δ* and *rad9Δ*. Spores (44 tetrads) from a yeast strain heterozygous for *cdc13-1*, *sgs1Δ*, *exo1Δ, sae2Δ* and *rad9Δ* were dissected onto YPD agar plate and the plates were incubated at 23°C for five days before being photographed. Five tetrads (labelled 1–5) are shown. The genotypes of the individual spores are indicated in the diagram at the bottom of the photograph, genotypes in brackets are inferred (s1 =  *sgs1Δ*, e1 =  *exo1Δ*, 9 =  *rad9Δ*, s2 =  *sae2Δ*, 13 =  *cdc13-1*).
(0.59 MB PDF)

Figure S7 Checkpoint response to telomere uncapping in *cdc13-1 exo1Δ* and *cdc13-1 sgs1Δ exo1Δ* strains. Yeast strains of the indicated genotypes (all with *cdc13-1 cdc15-2 bar1* mutations) were arrested in G1 at 23°C with α factor and released into 36°C, cells were collected at the indicated time points, and scored for the percentage of cells arrested in medial nuclear division and late nuclear division using DAPI staining.
(0.59 MB PDF)

Figure S8 Resection of uncapped telomeres in the absence of Sgs1, Exo1 and Rad9. A series of yeast strains with the indicated genotypes (with *cdc13-1 cdc15-2 bar1* mutations) were arrested in G1 at 23°C and released into 36°C to induce telomere uncapping, the amount of ssDNA at both TG and AC strands at two repetitive telomeric loci, *Y'600* and *Y'5000* and three single copy loci, *YER188W*, *YER186C* and *PDA1* were measured by QAOS as described in Figure 3. The values plotted are the mean value ± SD.
(1.32 MB PDF)

Figure S9 Resection of uncapped telomeres at *Y'5000* locus in *cdc13-1 exo1Δ* and *cdc13-1 sgs1Δ exo1Δ* strains. *exo1Δ* and *sgs1Δ exo1Δ* strains (with *cdc13-1 cdc15-2 bar1* mutations) were arrested in G1 at 23°C and released into 36°C to induce telomere uncapping, the amount of ssDNA at telomeric locus *Y'5000* were measured by QAOS as described in Figure 3. The values plotted are the mean value ± SD.
(0.51 MB PDF)

Figure S10 Genotypes of spores from a yeast strain heterozygous for *cdc13Δ*, *sgs1Δ*, *exo1Δ* and *rad9Δ*. Spores from a yeast strain heterozygous for *cdc13Δ*, *sgs1Δ*, *exo1Δ* and *rad9Δ* were dissected onto YPD agar plate and the plates were incubated at 23°C for five days before being photographed. Four tetrads (labelled 1-4) are shown. The genotypes of the spores were tested by patching and replica-plating onto relevant drop-out/antibiotic plates. The genotypes of the individual spores are indicated in the diagram at the bottom of the photograph, genotypes in brackets are inferred (full or partial) (s1 =  *sgs1Δ*, e1 =  *exo1Δ*, 9 =  *rad9Δ*, 13 =  *cdc13Δ*).
(0.53 MB PDF)

Figure S11 Senescence test of *cdc13Δ rad9Δ sgs1Δ exo1Δ* strains. Yeast strains of the indicated genotype were streaked onto YPD agar plates and the plates were incubated at 23°C for four days. The strains were then photographed and restreaked onto another YPD plate. This cycle is repeated for eight times (p = passage).
(5.81 MB PDF)

Figure S12 Telomere structure alteration in *cdc13Δ rad9Δ sgs1Δ exo1Δ* survivors. DNA was purified from *cdc13Δ sgs1Δ exo1Δ rad9Δ* (after patching, genotype testing and restreaking on YPD) or control strains following further incubation in liquid culture for 48 hours at 23°C. Southern blots were performed as in Figure 1C.
(0.67 MB PDF)

Table S1 Ratio of *cdc13Δ/CDC13* spores in *rad9Δ sgs1Δ exo1Δ* background. Spores from a yeast strain heterozygous for *cdc13Δ*, *sgs1Δ*, *exo1Δ* and *rad9Δ* were either dissected (154 tetrads) or treated for random sporulation. The genotypes of the spores were tested by patching and replica-plating onto relevant drop-out/antibiotic plates.
(0.64 MB PDF)

Table S2 Yeast strains used in the study.
(2.43 MB PDF)
