# Supplementary figures and images for: Survival and Growth of Yeast without Telomere Capping by Cdc13 in the Absence of Sgs1, Exo1, and Rad9
Source: PLoS Genet. 2010 Aug 19;6(8):e1001072. doi: 10.1371/journal.pgen.1001072 (PMC2924318; doi:10.1371/journal.pgen.1001072)

Figure S1

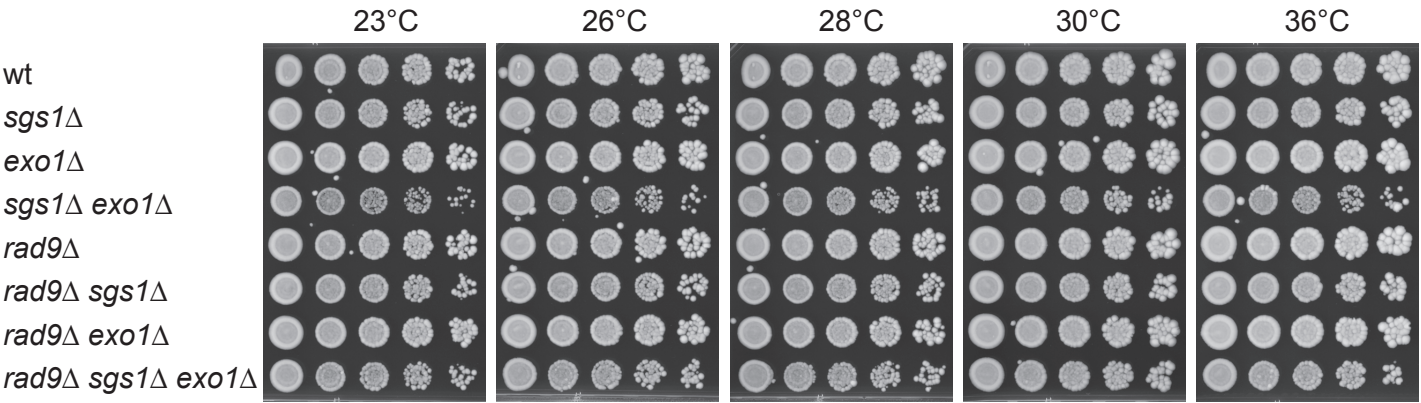

Supplement: Figure S1 — Growth of CDC13 strains at different temperatures. Serial dilutions of yeast strains with the indicated genotypes and growing at 23°C were spotted onto the same YPD agar plates in Figure 1A and incubated at the indicated temperatures for four days before being photographed. (1.98 MB PDF) [file pgen.1001072.s001.pdf]

Figure S2

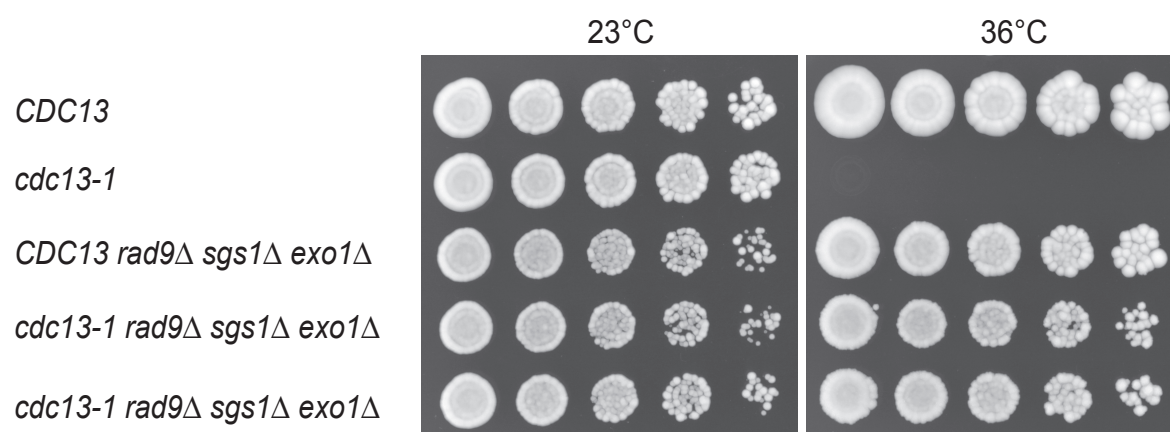

Supplement: Figure S2 — cdc13-1 rad9Δ sgs1Δ exo1Δ strains show little sensitivity to telomere uncapping at 36°C. Serial dilutions of yeast strains with the indicated genotypes and growing at 23°C were spotted onto YPD agar plates and incubated at the indicated temperatures for three days before being photographed. (3.90 MB PDF) [file pgen.1001072.s002.pdf]

Figure S3

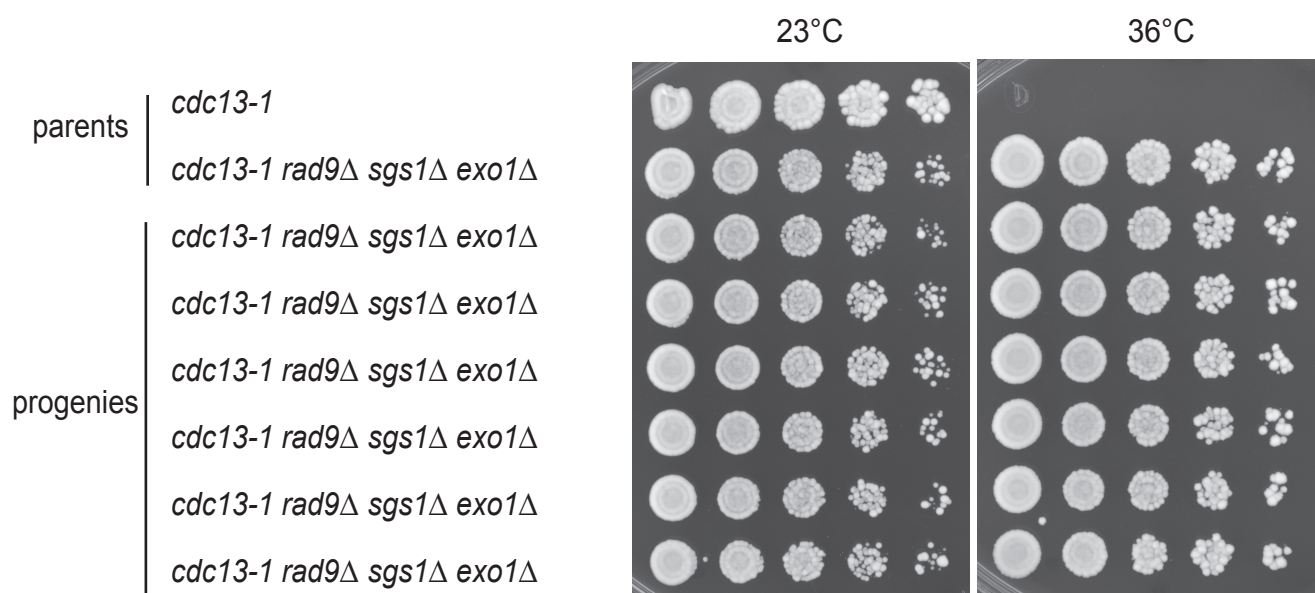

Supplement: Figure S3 — Growth of cdc13-1 rad9Δ sgs1Δ exo1Δ strains at 36°C is not due to another mutation. Serial dilutions of yeast strains with the indicated genotypes and growing at 23°C were spotted onto YPD agar plates and incubated at the indicated temperatures for three days before being photographed. (5.75 MB PDF) [file pgen.1001072.s003.pdf]

# Figure S4

**A**

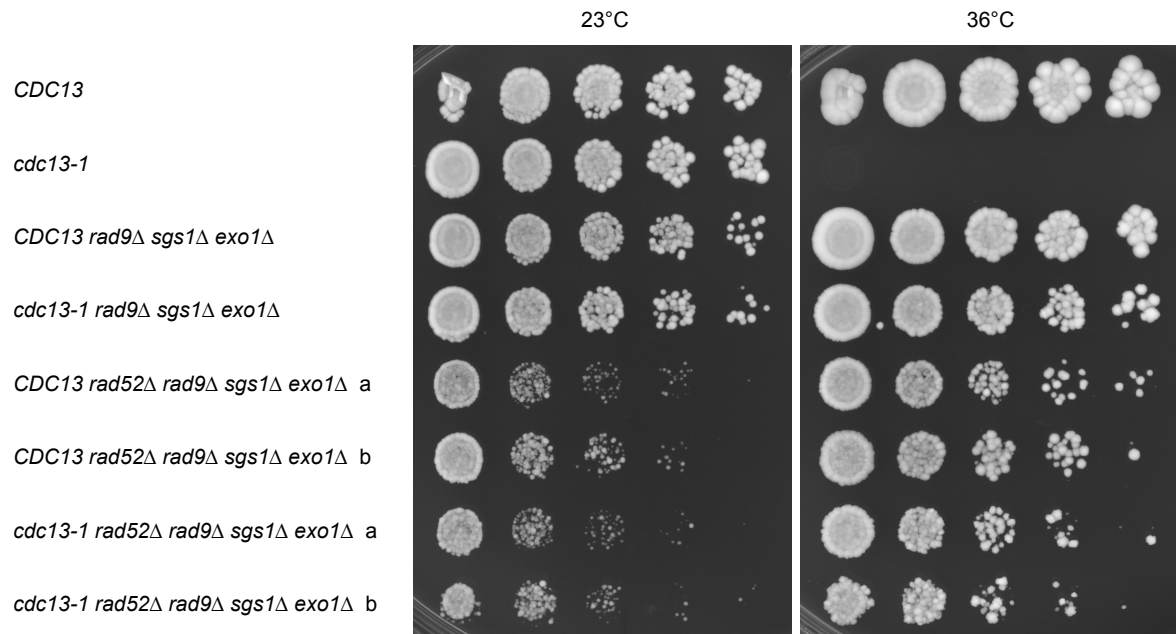

**B**

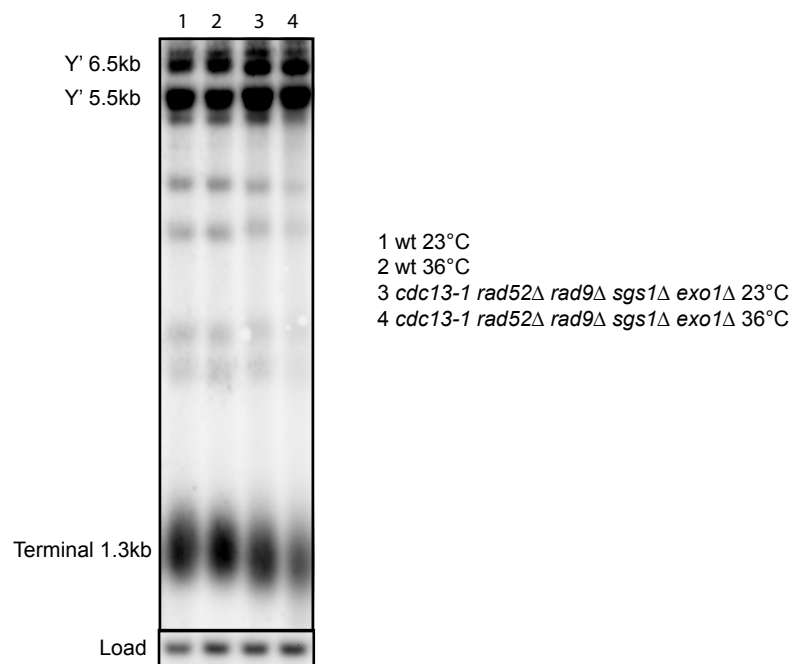

Supplement: Figure S4 — Growth of cdc13-1 rad9Δ sgs1Δ exo1Δ strains at 36°C is not dependant on Rad52. (A) Serial dilutions of yeast strains with the indicated genotypes and growing at 23°C were spotted onto YPD agar plates and incubated at the indicated temperatures for three days before being photographed. (B) DNA was purified from the strains with the indicated genotypes following further incubation in liquid culture overnight at 23°C or 36°C. Southern blots were performed as in Figure 1C. (5.79 MB PDF) [file pgen.1001072.s004.pdf]

Figure S5

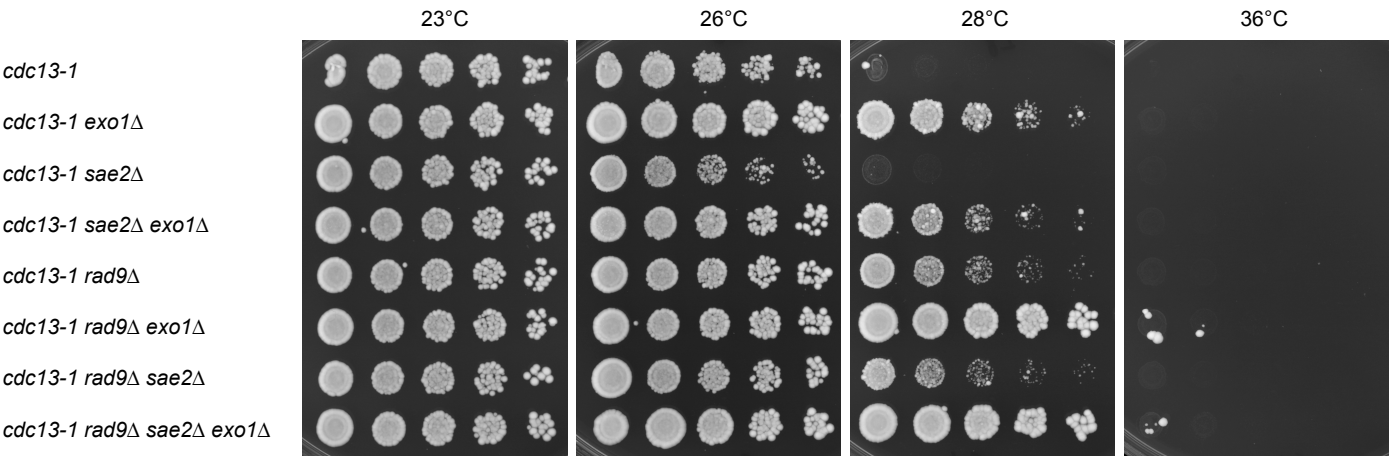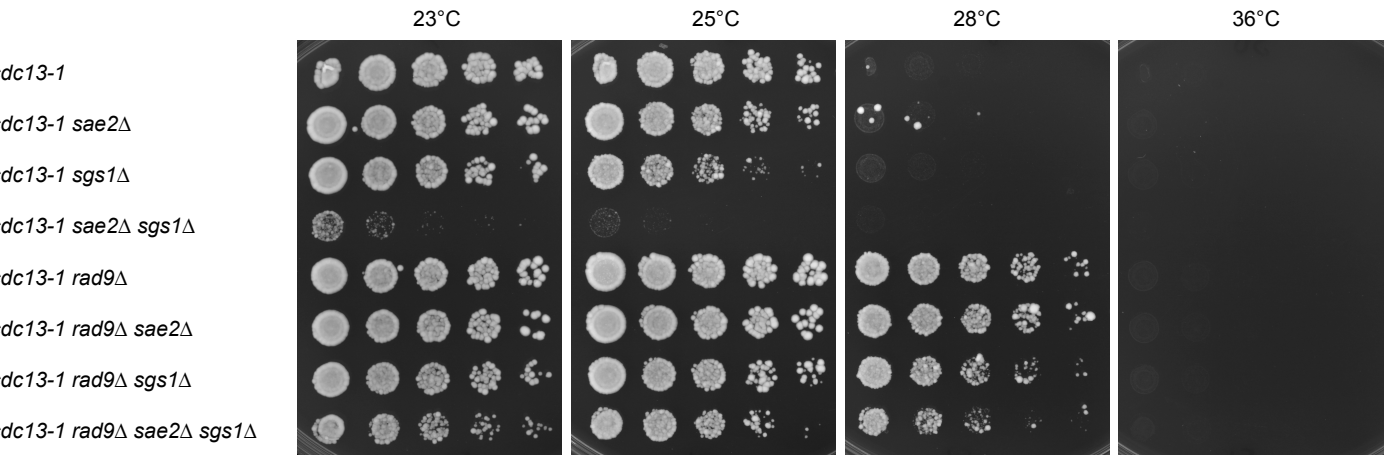

Supplement: Figure S5 — Genetic interactions of sae2Δ with sgs1Δ, exo1Δ, rad9Δ and cdc13-1. Serial dilutions of yeast strains with the indicated genotypes and growing at 23°C were spotted onto YPD agar plates and incubated at the indicated temperatures for three days before being photographed. The top panel show genetic interaction of sae2Δ with exo1Δ, rad9Δ and cdc13-1 whereas the bottom panel show genetic interaction of sae2Δ with sgs1Δ, rad9Δ and cdc13-1. (9.47 MB PDF) [file pgen.1001072.s005.pdf]

Figure S6

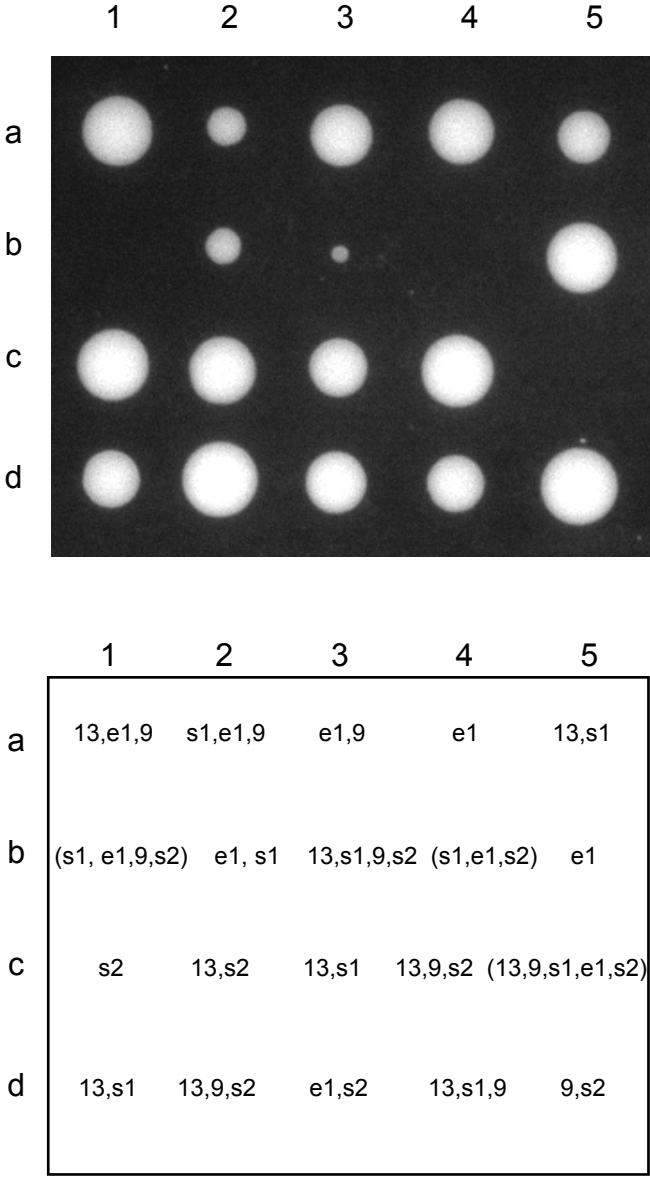

Supplement: Figure S6 — Genotypes of spores from a yeast strain heterozygous for cdc13-1, sgs1Δ, exo1Δ, sae2Δ and rad9Δ. Spores (44 tetrads) from a yeast strain heterozygous for cdc13-1, sgs1Δ, exo1Δ, sae2Δ and rad9Δ were dissected onto YPD agar plate and the plates were incubated at 23°C for five days before being photographed. Five tetrads (labelled 1–5) are shown. The genotypes of the individual spores are indicated in the diagram at the bottom of the photograph, genotypes in brackets are inferred (s1 = sgs1Δ, e1 = exo1Δ, 9 = rad9Δ, s2 = sae2Δ, 13 = cdc13-1). (0.59 MB PDF) [file pgen.1001072.s006.pdf]

Figure S7

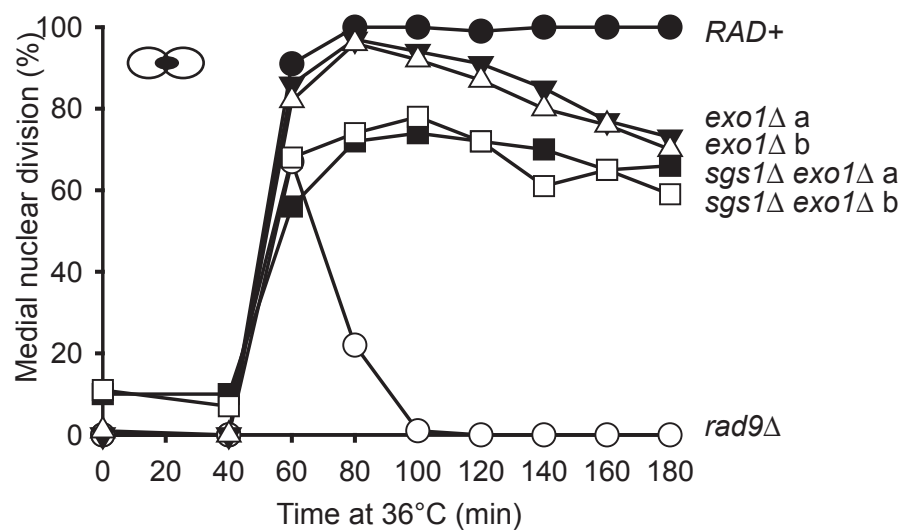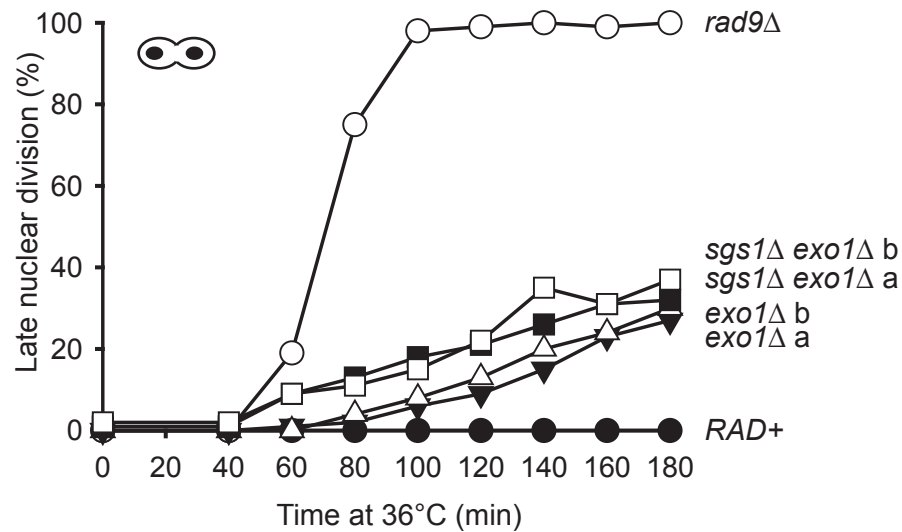

Supplement: Figure S7 — Checkpoint response to telomere uncapping in cdc13-1 exo1Δ and cdc13-1 sgs1Δ exo1Δ strains. Yeast strains of the indicated genotypes (all with cdc13-1 cdc15-2 bar1 mutations) were arrested in G1 at 23°C with α factor and released into 36°C, cells were collected at the indicated time points, and scored for the percentage of cells arrested in medial nuclear division and late nuclear division using DAPI staining. (0.59 MB PDF) [file pgen.1001072.s007.pdf]

# Figure S8

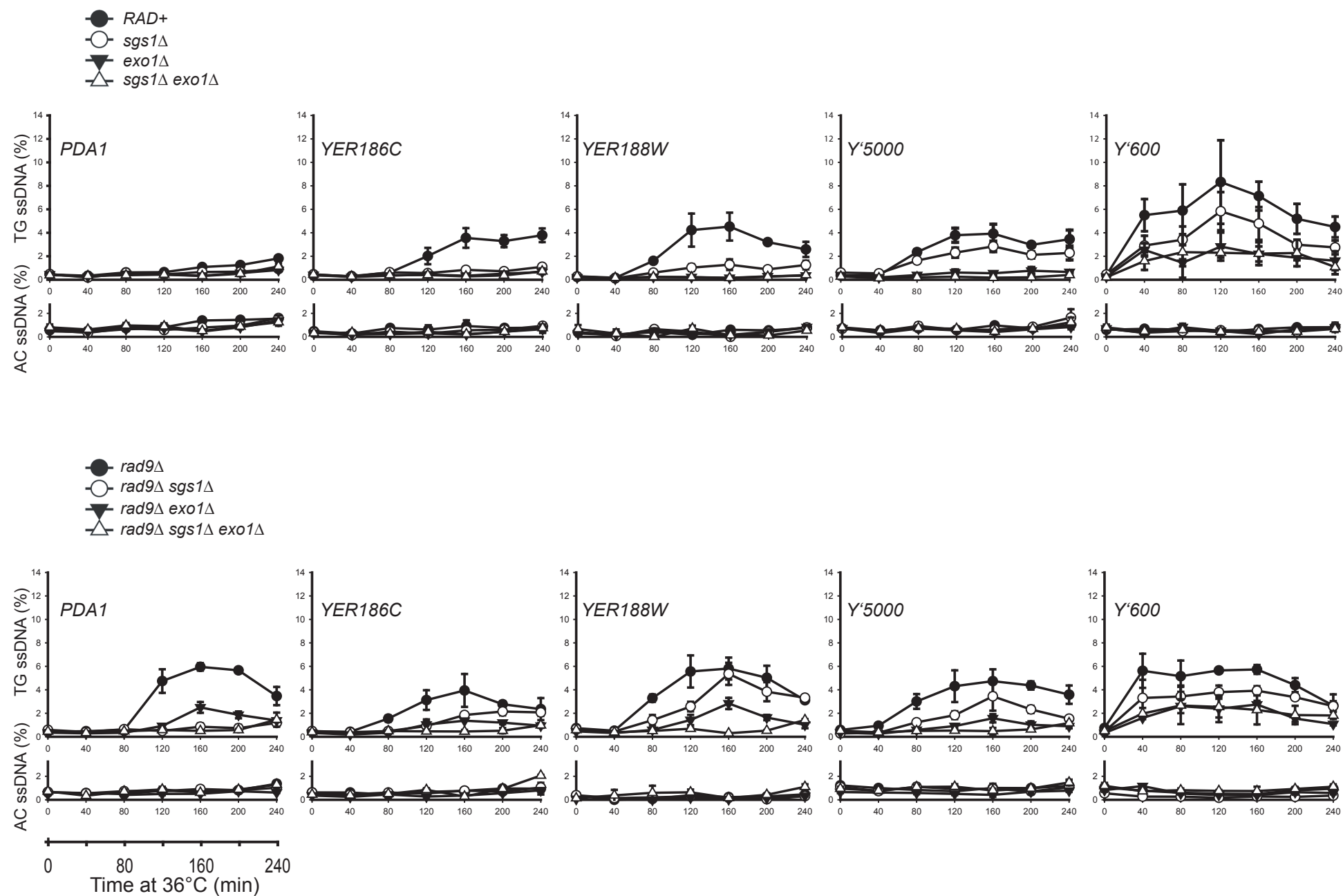

Supplement: Figure S8 — Resection of uncapped telomeres in the absence of Sgs1, Exo1 and Rad9. A series of yeast strains with the indicated genotypes (with cdc13-1 cdc15-2 bar1 mutations) were arrested in G1 at 23°C and released into 36°C to induce telomere uncapping, the amount of ssDNA at both TG and AC strands at two repetitive telomeric loci, Y'600 and Y'5000 and three single copy loci, YER188W, YER186C and PDA1 were measured by QAOS as described in Figure 3. The values plotted are the mean value ± SD. (1.32 MB PDF) [file pgen.1001072.s008.pdf]

Figure S9

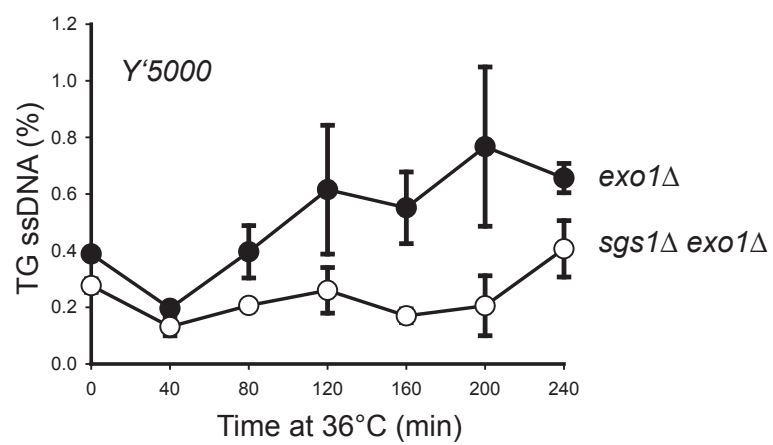

Supplement: Figure S9 — Resection of uncapped telomeres at Y'5000 locus in cdc13-1 exo1Δ and cdc13-1 sgs1Δ exo1Δ strains. exo1Δ and sgs1Δ exo1Δ strains (with cdc13-1 cdc15-2 bar1 mutations) were arrested in G1 at 23°C and released into 36°C to induce telomere uncapping, the amount of ssDNA at telomeric locus Y'5000 were measured by QAOS as described in Figure 3. The values plotted are the mean value ± SD. (0.51 MB PDF) [file pgen.1001072.s009.pdf]

Figure S10

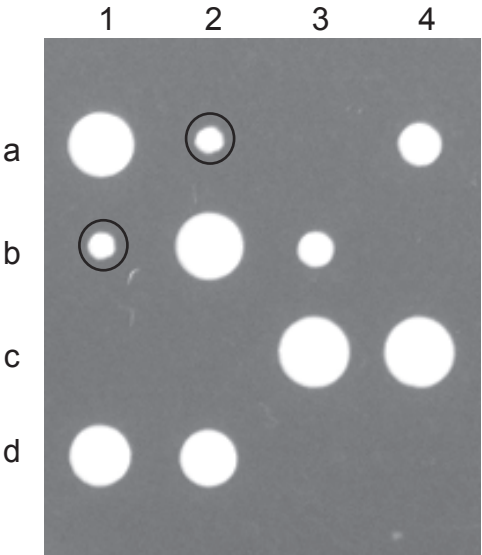

○ *cdc13Δ rad9Δ sgs1Δ exo1Δ*

|   | 1          | 2          | 3       | 4    |
|---|------------|------------|---------|------|
| a | e1         | s1,e1,9,13 | (13)    | s1,9 |
| b | s1,e1,9,13 | e1         | s1,e1,9 | (13) |
| c | (13)       | (13)       | WT      | e1,9 |
| d | s1,9       | s1,9       | (13)    | (13) |

Supplement: Figure S10 — Genotypes of spores from a yeast strain heterozygous for cdc13Δ, sgs1Δ, exo1Δ and rad9Δ. Spores from a yeast strain heterozygous for cdc13Δ, sgs1Δ, exo1Δ and rad9Δ were dissected onto YPD agar plate and the plates were incubated at 23°C for five days before being photographed. Four tetrads (labelled 1-4) are shown. The genotypes of the spores were tested by patching and replica-plating onto relevant drop-out/antibiotic plates. The genotypes of the individual spores are indicated in the diagram at the bottom of the photograph, genotypes in brackets are inferred (full or partial) (s1 = sgs1Δ, e1 = exo1Δ, 9 = rad9Δ, 13 = cdc13Δ). (0.53 MB PDF) [file pgen.1001072.s010.pdf]

Figure S11

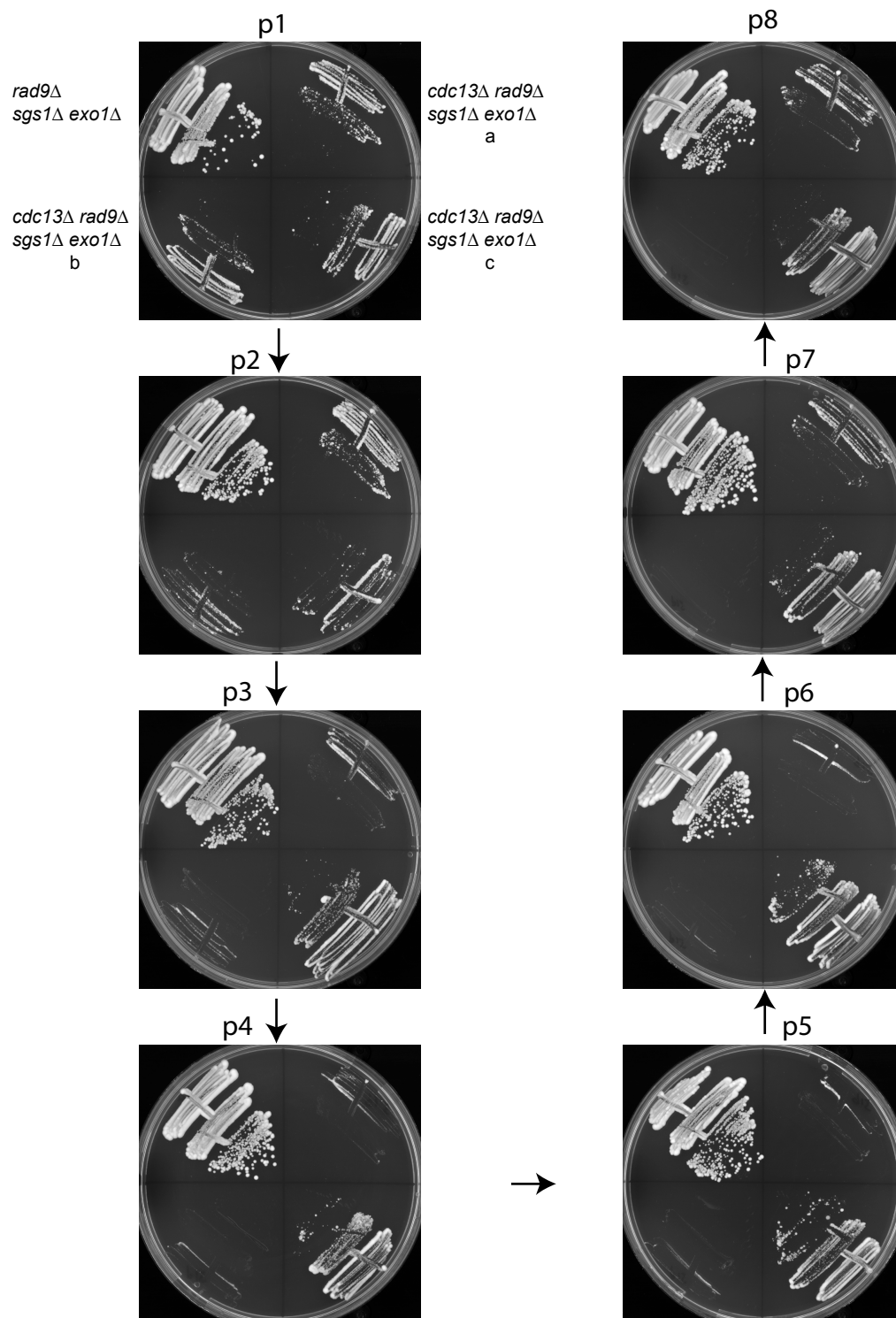

Supplement: Figure S11 — Senescence test of cdc13Δ rad9Δ sgs1Δ exo1Δ strains. Yeast strains of the indicated genotype were streaked onto YPD agar plates and the plates were incubated at 23°C for four days. The strains were then photographed and restreaked onto another YPD plate. This cycle is repeated for eight times (p = passage). (5.81 MB PDF) [file pgen.1001072.s011.pdf]

Figure S12

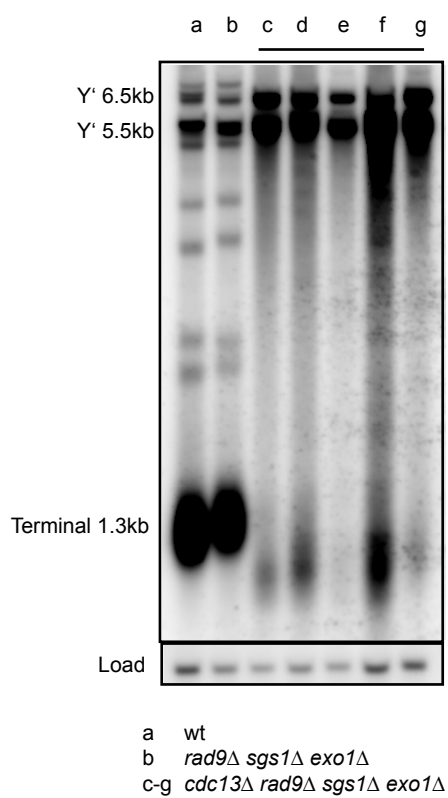

Supplement: Figure S12 — Telomere structure alteration in cdc13Δ rad9Δ sgs1Δ exo1Δ survivors. DNA was purified from cdc13Δ sgs1Δ exo1Δ rad9Δ (after patching, genotype testing and restreaking on YPD) or control strains following further incubation in liquid culture for 48 hours at 23°C. Southern blots were performed as in Figure 1C. (0.67 MB PDF) [file pgen.1001072.s012.pdf]
