# Supplementary material for: Survival and Growth of Yeast without Telomere Capping by Cdc13 in the Absence of Sgs1, Exo1, and Rad9
Source: PLoS Genet. 2010 Aug 19;6(8):e1001072. doi: 10.1371/journal.pgen.1001072 (PMC2924318; doi:10.1371/journal.pgen.1001072)
Supplement: Table S1 — Ratio of cdc13Δ/CDC13 spores in rad9Δ sgs1Δ exo1Δ background. Spores from a yeast strain heterozygous for cdc13Δ, sgs1Δ, exo1Δ and rad9Δ were either dissected (154 tetrads) or treated for random sporulation. The genotypes of the spores were tested by patching and replica-plating onto relevant drop-out/antibiotic plates. (0.64 MB PDF) [file pgen.1001072.s013.pdf]

Table S1 Ratio of *cdc13Δ/CDC13* spores in *rad9Δ sgs1Δ exo1Δ* background

	Viable spores examined	Number of *CDC13 sgs1Δ exo1Δ rad9Δ* spores observed	Number of *cdc13Δ sgs1Δ exo1Δ rad9Δ* spores observed	Ratio of *cdc13Δ/CDC13*
Tetrad dissection	325	38 (expected=38.5)	29 (expected=38.5)	0.76
Random sporulation	400	46 (expected=44.4)	47 (expected=44.4)	1.02
