# Supplementary material for: Survival and Growth of Yeast without Telomere Capping by Cdc13 in the Absence of Sgs1, Exo1, and Rad9
Source: PLoS Genet. 2010 Aug 19;6(8):e1001072. doi: 10.1371/journal.pgen.1001072 (PMC2924318; doi:10.1371/journal.pgen.1001072)
Supplement: Table S2 — Yeast strains used in the study. (2.43 MB PDF) [file pgen.1001072.s014.pdf]

Table S2 Yeast strains

Strain	Genotype	Figures
DLY640	MATa ade2-1 trp1-1 can1-100 leu2-3,112 his3-11,15 ura3 GAL+ psi+ ssd1-d2 RAD5	1C,1D,4B, 5B,S1,S2
DLY4557	MATa cdc13-1int ade2-1 trp1-1 can1-100 leu2-3,112 his3-11,15 ura3 GAL+ psi+ ssd1-d2 RAD5	1A,4A,S2, S4
DLY4719	MATa cdc13-1int ade2-1 trp1-1 can1-100 leu2-3,112 his3-11,15 ura3 GAL+ psi+ ssd1-d2 RAD5 sgs1Δ::KanMX	1A
DLY4921	MATa cdc13-1int ade2-1 trp1-1 can1-100 leu2-3,112 his3-11,15 ura3 GAL+ psi+ ssd1-d2 RAD5 exo1::LEU2	1A
DLY4910	MATa cdc13-1int ade2-1 trp1-1 can1-100 leu2-3,112 his3-11,15 ura3 GAL+ psi+ ssd1-d2 RAD5 sgs1Δ::KanMX exo1Δ::LEU2	1A
DLY4647	MATa cdc13-1int ade2-1 trp1-1 can1-100 leu2-3,112 his3-11,15 ura3 GAL+ psi+ ssd1-d2 RAD5 rad9Δ::HIS3	1A
DLY4720	MATa cdc13-1int ade2-1 trp1-1 can1-100 leu2-3,112 his3-11,15 ura3 GAL+ psi+ ssd1-d2 RAD5 rad9Δ::HIS3 sgs1Δ::KanMX	1A
DLY4922	MATa cdc13-1int ade2-1 trp1-1 can1-100 leu2-3,112 his3-11,15 ura3 GAL+ psi+ ssd1-d2 RAD5 rad9Δ::HIS3 exo1Δ::LEU2	1A
DLY4912-4914	MATa/α cdc13-1int ade2-1 trp1-1 can1-100 leu2-3,112 his3-11,15 ura3 GAL+ psi+ ssd1-d2 RAD5 rad9Δ::HIS3 sgs1::KanMX exo1Δ::LEU2	1A,1C,1D, 4A,4B,S2, S3,S4
DLY5275-5280	MATa/α cdc13-1int ade2-1 trp1-1 can1-100 leu2-3,112 his3-11,15 ura3 GAL+ psi+ ssd1-d2 RAD5 rad9Δ::HIS3 sgs1::KanMX exo1Δ::LEU2	S3
DLY4918	MATa ade2-1 trp1-1 can1-100 leu2-3,112 his3-11,15 ura3 GAL+ psi+ ssd1-d2 RAD5 rad9Δ::HIS3 sgs1::KanMX exo1Δ::LEU2	1D,4A,S1, S2,S4,S12
DLY3926	MATa cdc13-1int ade2-1 trp1-1 can1-100 leu2-3,112 his3-11,15 ura3 GAL+ psi+ ssd1-d2 RAD5 pDL17	S3
DLY1628	MATa ade2-1 trp1-1 can1-100 leu2-3,112 his3-11,15 ura3 GAL+ psi+ ssd1-d2 RAD5 tlc1Δ::HIS3 p::URA3::TLC1	4
DLY5260/ 5261	MATa cdc13-1int ade2-1 trp1-1 can1-100 leu2-3,112 his3-11,15 ura3 GAL+ psi+ ssd1-d2 RAD5 cdc15-2 bar1	2,3,S7,S8, S9
DLY5123/ 5262	MATa cdc13-1int ade2-1 trp1-1 can1-100 leu2-3,112 his3-11,15 ura3 GAL+ psi+ ssd1-d2 RAD5 cdc15-2 bar1 sgs1Δ::KanMX	2,3,S7,S8, S9
DLY5263/ 5264	MATa cdc13-1int ade2-1 trp1-1 can1-100 leu2-3,112 his3-11,15 ura3 GAL+ psi+ ssd1-d2 RAD5 cdc15-2 bar1 rad9Δ::HIS3	2,3,S7,S8, S9
DLY5265/ 5266	MATa cdc13-1int ade2-1 trp1-1 can1-100 leu2-3,112 his3-11,15 ura3 GAL+ psi+ ssd1-d2 RAD5 cdc15-2 bar1 exo1Δ::LEU2	2,3,S7,S8, S9
DLY5269/ 5270	MATa cdc13-1int ade2-1 trp1-1 can1-100 leu2-3,112 his3-11,15 ura3 GAL+ psi+ ssd1-d2 RAD5 cdc15-2 bar1 sgs1Δ::KanMX exo1Δ::LEU2	2,3,S7,S8, S9
DLY5267/ 5268	MATa cdc13-1int ade2-1 trp1-1 can1-100 leu2-3,112 his3-11,15 ura3 GAL+ psi+ ssd1-d2 RAD5 cdc15-2 bar1 rad9Δ::HIS3 sgs1Δ::KanMX	2,3,S7,S8, S9
DLY5131/ 5132	MATa cdc13-1int ade2-1 trp1-1 can1-100 leu2-3,112 his3-11,15 ura3 GAL+ psi+ ssd1-d2 RAD5 cdc15-2 bar1 rad9Δ::HIS3 exo1Δ::LEU2	2,3,S7,S8, S9
DLY5272/ 5273	MATa cdc13-1int ade2-1 trp1-1 can1-100 leu2-3,112 his3-11,15 ura3 GAL+ psi+ ssd1-d2 RAD5 cdc15-2 bar1 rad9Δ::HIS3 sgs1Δ::KanMX exo1Δ::LEU2	2,3,S7,S8, S9
DDY337	MATa/Matα ade2-1/ade2-1 trp1-1/trp1-1 can1-100/can1-100 leu2-3,112/leu2-3,112 his3-11,15/his3-11,15 ura3/ura3 GAL+/GAL+ psi+/psi+ ssd1-d2/ssd1-d2 RAD5/RAD5 cdc13Δ::hphMX/CDC13 sgs1::KanMX/SGS1 exo1Δ::leu2/EXO1 rad9Δ::HIS3/RAD9	5,S10
DLY4721	MATa ade2-1 trp1-1 can1-100 leu2-3,112 his3-11,15 ura3 GAL+ psi+ ssd1-d2 RAD5 sgs1Δ::KanMX	S1
DLY1273	MATα ade2-1 trp1-1 can1-100 leu2-3,112 his3-11,15 ura3 GAL+ psi+ ssd1-d2 RAD5 exo1::LEU2	S1
DLY4915	MATa ade2-1 trp1-1 can1-100 leu2-3,112 his3-11,15 ura3 GAL+ psi+ ssd1-d2 RAD5 sgs1::KanMX exo1Δ::LEU2	S1
DLY4724	MATa ade2-1 trp1-1 can1-100 leu2-3,112 his3-11,15 ura3 GAL+ psi+ ssd1-d2 RAD5 rad9Δ::HIS3 sgs1::KanMX	S1
DLY1698	MATa ade2-1 trp1-1 can1-100 leu2-3,112 his3-11,15 ura3 GAL+ psi+ ssd1-d2 RAD5 rad9Δ::HIS3 exo1Δ::LEU2	S1
DLY6189	MATα cdc13-1int ade2-1 trp1-1 can1-100 leu2-3,112 his3-11,15 ura3 GAL+ psi+ ssd1-d2 RAD5 sae2:TRP1	S5
DLY6190	MATa cdc13-1int ade2-1 trp1-1 can1-100 leu2-3,112 his3-11,15 ura3 GAL+ psi+ ssd1-d2 RAD5 sae2:TRP1 exo1Δ::LEU2	S5
DLY6192	MATa cdc13-1int ade2-1 trp1-1 can1-100 leu2-3,112 his3-11,15 ura3 GAL+ psi+ ssd1-d2 RAD5 sae2:TRP1 rad9Δ::HIS3	S5
DLY6194	MATα cdc13-1int ade2-1 trp1-1 can1-100 leu2-3,112 his3-11,15 ura3 GAL+ psi+ ssd1-d2 RAD5 sae2:TRP1 rad9Δ::HIS3 sgs1Δ::KanMX	S5
DLY6198	MATa cdc13-1int ade2-1 trp1-1 can1-100 leu2-3,112 his3-11,15 ura3 GAL+ psi+ ssd1-d2 RAD5 sae2:TRP1 rad9Δ::HIS3 exo1Δ::LEU2	S5
DLY6221	MATa cdc13-1int ade2-1 trp1-1 can1-100 leu2-3,112 his3-11,15 ura3 GAL+ psi+ ssd1-d2 RAD5 sae2:TRP1 sgs1Δ::KanMX	S5
DLY5886/ 5887	MATa/α ade2-1 trp1-1 can1-100 leu2-3,112 his3-11,15 ura3 GAL+ psi+ ssd1-d2 RAD5 rad9Δ::HIS3 sgs1::KanMX exo1Δ::LEU2 rad52Δ::TRP1	S4
DLY5888/ 5889	MATa/α cdc13-1int ade2-1 trp1-1 can1-100 leu2-3,112 his3-11,15 ura3 GAL+ psi+ ssd1-d2 RAD5 rad9Δ::HIS3 sgs1::KanMX exo1Δ::LEU2 rad52Δ::TRP1	S4
